# Identification and validation of a two-gene expression index for subtype classification and prognosis in Diffuse Large B-Cell Lymphoma

**DOI:** 10.1038/srep10006

**Published:** 2015-05-05

**Authors:** Qinghua Xu, Cong Tan, Shujuan Ni, Qifeng Wang, Fei Wu, Fang Liu, Xun Ye, Xia Meng, Weiqi Sheng, Xiang Du

**Affiliations:** 1 Department of Oncology, Shanghai Medical College, Fudan University, Shanghai, China; 2 Department of Pathology, Fudan University Shanghai Cancer Center, Shanghai, China; 3 Institute of Pathology, Fudan University, Shanghai, China; 4 Fudan University Shanghai Cancer Center – Institut Mérieux Laboratory, Shanghai, China; 5 bioMérieux (Shanghai) Company Limited, Shanghai, China

## Abstract

The division of diffuse large B-cell lymphoma (DLBCL) into germinal center B-cell-like (GCB) and activated B-cell-like (ABC) subtypes based on gene expression profiling has proved to be a landmark in understanding the pathogenesis of the disease. This study aims to identify a novel biomarker to facilitate the translation of research into clinical practice. Using a training set of 350 patients, we identified a two-gene expression signature, “LIMD1-MYBL1 Index”, which is significantly associated with cell-of-origin subtypes and clinical outcome. This two-gene index was further validated in two additional dataset. Tested against the gold standard method, the LIMD1-MYBL1 Index achieved 81% sensitivity, 89% specificity for ABC group and 81% sensitivity, 87% specificity for GCB group. The ABC group had significantly worse overall survival than the GCB group (hazard ratio = 3.5, *P* = 0.01). Furthermore, the performance of LIMD1-MYBL1 Index was satisfactory compared with common immunohistochemical algorithms. Thus, the LIMD1-MYBL1 Index had considerable clinical value for DLBCL subtype classification and prognosis. Our results might prompt the further development of this two-gene index to a simple assay amenable to routine clinical practice.

Diffuse large B-cell lymphoma (DLBCL) is the most common lymphoma worldwide, accounting for nearly 30 to 40% of non-Hodgkin’s lymphoma cases. DLBCL is highly heterogeneous from both morphological and clinical standpoints. The standard therapy for patients with DLBCL is Rituximab^®^ combined with cyclophosphamide, doxorubicin, vincristine, and prednisone (R-CHOP), and this regimen results in a long-term disease-free survival rate of approximately 50%^1^. The International Prognostic Index (IPI) is the current standard approach to estimate the prognoses of DLBCL patients. The IPI stratifies DLBCL patients into four risk groups (low, low-intermediate, high-intermediate, and high). However, within each of these IPI risk groups, there are considerable differences with respect to outcome, suggesting that there are underlying biological heterogeneities that are not accounted for by the traditional clinical parameters. Through gene expression profiling, Alizadeh *et al.* identified two major cell-of-origin (COO) phenotypes with distinct prognoses: the favorable germinal centre B-cell-like (GCB) and the unfavorable activated B-cell-like (ABC) subtypes^2^. The distinct biological and clinical features of these subtypes have been independently validated^3^, and therefore, these two groupings are recognized as DLBCL subtypes in the current World Health Organization classification^4^.

With the rapid evolution of microarray technology over the last decade, there have been multiple follow-up studies performed in this field using standardized genome-wide microarrays^5^,^6^,^7^,^8^,^9^,^10^,^11^,^12^, which have generated large volumes of gene expression data. Given the vast amounts of publicly available microarray data, the integrative analysis of microarrays, in which data from multiple studies are combined to increase the sample size and avoid laboratory-specific bias, has the potential to yield new biological insights that are not possible from a single study, as already demonstrated for prostate and other cancers^13^. Here, we describe an integrative analysis leading to identification and validation of a novel biomarker for both subtype classification and survival prediction in DLBCL.

## Results

### The LIMD1-MYBL1 Index was associated with the COO subtypes in DLBCL

In this study, we included three gene expression dataset for biomarker discovery and validation. The DLBCL-1 dataset were used as a training set to identify gene expression signatures, and the DLBCL-2 and DLBCL-3 dataset were used as independent test sets for validation purpose. Details of study designs and sample characteristics are provided in Table 1.

**Figure 1:**
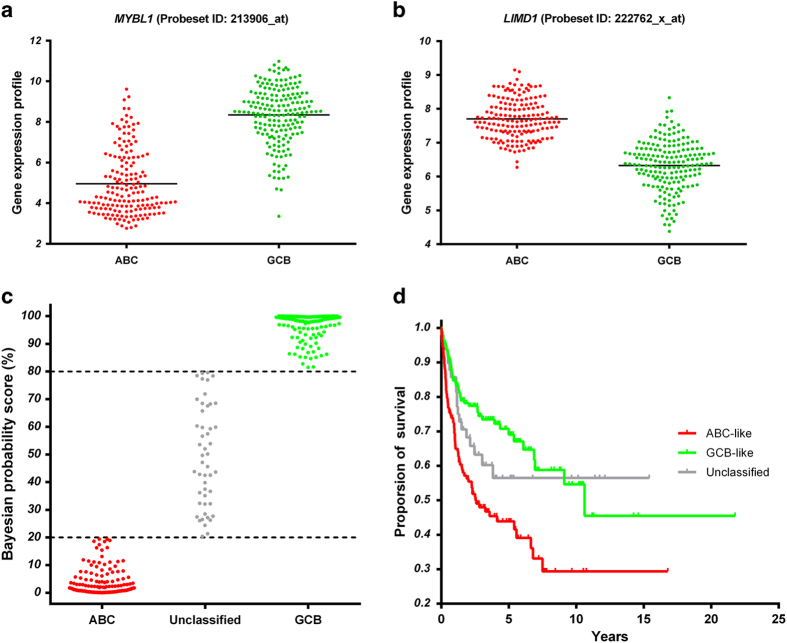
The LIMD1-MYBL1 Index was significantly associated with COO subtypes and clinical outcomes in DLBCL-1 (**a**) The gene *MYBL1* (v-myb myeloblastosis viral oncogene homolog (avian)-like 1) was highly expressed in GCB subtype compared with ABC subtype (*P* = 1.5E-64). (**b**) The gene *LIMD1* (LIM domains containing 1) was significantly over-expressed in ABC subtype compared with GCB subtype (*P* = 5.7E-58). (**c**) For each patient, a Bayesian probability score was estimated. A sample is classified as ABC or GCB subtype if the probability that it belongs to the ABC or GCB subgroup is greater than 80%; otherwise it is considered as unclassified subtype. (**d**) The Kaplan-Meier estimates of overall survival for 350 patients classified by the LIMD1-MYBL1 Index. GCB group had significantly higher overall survival rates compared with ABC group (*P* < 0.001).

The DLBCL-1 cohort included 167 ABC and 183 GCB DLBCL patients according to the gold standard method described by Wright *et al.*^5^. We performed a two-class unpaired t-test to select genes that were differentially expressed between ABC and GCB subgroups, and then ranked the genes in descending order according to their statistical significance. The top two probesets were particularly interesting. One probeset, “213906_at”, which targets the gene *MYBL1* (v-myb myeloblastosis viral oncogene homolog (avian)-like 1), exhibited a 10-fold higher expression level in the GCB group compared with the ABC group (*P* = 1.5E-64; Fig. 1a). Sensitivity versus 1-specificity was plotted to construct a Receiver Operating Characteristic (ROC) curve, and a good discrimination between the two groups was observed, with an Area Under Curve (AUC) of 0.93. In sharp contrast, the probeset “222762_x_at”, targeting the gene *LIMD1* (LIM domains containing 1), was significantly over-expressed in the ABC group compared with the GCB group (*P* = 5.7E-58; Fig. 1b). The discriminatory power measured by the AUC was 0.94.

Since *LIMD1* and *MYBL1* exhibited distinct expression patterns in ABC- and GCB- DLBCLs respectively, we integrated these two genes into a Bayesian classifier similar to the gold standard method^5^, and defined it as “LIMD1-MYBL1 Index”. For each patient, a probability score was estimated. A sample is classified as ABC or GCB subtype if the probability that it belongs to the ABC or GCB subgroup is greater than 80%; otherwise it is considered as unclassified type. Accordingly, the LIMD1-MYBL1 Index correctly classified 137 out of 167 ABC and 151 out of 183 GCB cases, resulting in 82% sensitivity, 86% specificity for ABC group and 83% sensitivity, 83% specificity for GCB group (Fig. 1c). The discriminatory power measured by AUC was further improved to 0.97.

### The LIMD1-MYBL1 Index was an independent factor for DLBCL prognosis

The most important test of the LIMD1-MYBL1 Index was the ability to predict clinical outcome. Overall survival rates were significantly different between the ABC and GCB subgroups classified by the LIMD1-MYBL1 Index (*P* < 0.001) (Fig. 1d). In univariate proportional hazards regression analysis, relative to GCB class as baseline, the hazard ratio for ABC class was 2.3 (95% CI: 1.5–3.2, *P* < 0.001). Then, multivariate analysis was performed to demonstrate whether the LIMD1-MYBL1 Index could provide additional prognostic information beyond the conventional clinical parameters, such as age, stage, ECOG performance status, LDH level and number of extranodal sites. In addition, the multivariate proportional hazards regression also included the treatment variable: CHOP *vs.* R-CHOP therapy. As shown in Table 2, the LIMD1-MYBL1 Index consistently contributed additional prognostic information independent of either summarized IPI score (hazard ratio = 2.1, *P* < 0.001) or individual IPI constituent factors (hazard ratio = 2.2, *P* < 0.001).

### Clinical validation of the LIMD1-MYBL1 Index in Chinese patients

**Figure 2:**
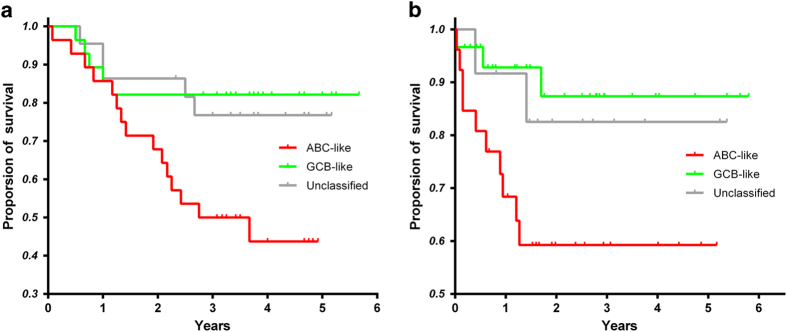
The subtype classification based on LIMD1-MYBL1 Index was significantly associated with patients’ survival (**a**) The Kaplan-Meier estimates of overall survival for 88 patients in DLBCL-2. (**b**) The Kaplan-Meier estimates of overall survival for 68 patients in DLBCL-3.

Since the gene expression-based COO subtypes was first described more than a decade ago^2^, most studies have been conducted with DLBCL patients from the Western population, and few data have been published concerning the application of the COO subtypes in the Chinese population. In this study, we performed gene expression profiling for 88 Chinese patients. With the LIMD1-MYBL1 Index, 32 were classified as ABC (36%), 34 as GCB (39%) and 22 as unclassified cases (25%). When considering the 69 cases designated as ABC or GCB by the gold standard method, the LIMD1-MYBL1 Index correctly classified 26 out of 32 ABC and 30 out of 37 GCB cases, resulting in 81% sensitivity, 89% specificity for ABC group and 81% sensitivity, 87% specificity for GCB group. Furthermore, the subtypes assigned by LIMD1-MYBL1 Index was significantly associated with clinical outcomes. Overall survival rates was significantly better for GCB group compared with ABC group (hazard ratio = 3.5, *P* < 0.02; Fig. 2a). The C-statistic was 0.68 and 0.66 for LIMD1-MYBL1 Index and gold standard method respectively, suggesting very comparable prognostic ability of two algorithms (*P* = 0.39). As shown in Table S1, there were no significant differences between the classified subgroups in terms of demographics and patient characteristics, indicating that the LIMD1-MYBL1 Index classification indeed provided additional prognostic information independent of routine clinical parameters.

### Comparison of LIMD1-MYBL1 Index with other COO classification methods

Scott *et al.*^12^ has recently described a Nanostring-based Lymph2Cx assay for COO assignment and compared their assay with several immunohistochemical (IHC) algorithms. The performance of Lymph2Cx assay and IHC-based algorithms were evaluated in an independent cohort of 68 cases, drawn from the Lenz *et al.* dataset^8^. According to gold standard method, the cohort consisted of 30 ABC, 28 GCB and 10 unclassified cases. In order to avoid data overfitting, we used the unselected samples from Lenz *et al.* dataset to train the Bayesian classifier and then evaluated the LIMD1-MYBL1 Index in the Scott *et al.* dataset. Of the 68 samples, 26 were classified as ABC (38%), 30 as GCB (44%) and 12 as unclassified cases (18%). When considering the 58 cases designated as ABC or GCB by the gold standard method, the LIMD1-MYBL1 Index incorrectly assigned 1 cases: an ABC case was assigned to GCB group. At 2%, this favorably compares with the 9%, 17% and 6% rates of misassignments by the Hans, Choi, and Tally IHC-based algorithms, respectively. The Lymph2Cx assay performed similarly as the LIMD1-MYBL1 Index with 2% rates of misassignments of ABC and GCB cases. When considering the whole dataset including ten unclassified cases, the Lymph2Cx assay showed higher classification accuracy compared with the LIMD1-MYBL1 Index, although not statistically significant (80.6% and 75%, respectively; *P* = 0.57). Outcomes of 68 patients were used to determine whether the COO assignments made by LIMD1-MYBL1 Index maintained the prognostic significance. Similarly to the gold standard method and Lymph2Cx assay, the LIMD1-MYBL1 Index defined ABC groups indeed had significantly worse overall survival than the GCB groups (*P* < 0.02, Fig. 2b). On the other hand, outcomes in the COO groups assigned by Hans, Choi and Tally algorithms were not significantly different in the same cohort^12^.

## Discussion

The division of DLBCL into GCB and ABC types based on gene expression profiling has proved to be a landmark in understanding the pathogenesis of this disease.^11^ However, due to the expense, technical constraints and the need for intensive bioinformatics analysis, the use of gene expression-based COO subtypes for routine clinical use is challenging. The translation of gene expression-based COO classification into IHC-based algorithms that classify samples on the basis of expression of subtype-related proteins has been difficult^14^. IHC-based algorithms have yield conflicting results, probably due to several methodological differences: lack of standardization of tissue fixation, antigen retrieval, staining protocols and cutoffs for designating positivity of expression^15^. These controversies limit the clinical utility of IHC-based algorithms.

In this study, we identified the LIMD1-MYBL1 Index as a novel biomarker for both subtype classification and survival prediction in DLBCL. This two-gene signature was further tested with additional validation dataset. We present here, for the first time to our knowledge, the verified prognostic utility of gene expression-based COO subtypes in Chinese DLBCL patients. Tested against the gold standard Affymetrix-based method, the LIMD1-MYBL1 Index achieved 81% sensitivity, 89% specificity for ABC group and 81% sensitivity, 87% specificity for GCB group. Overall survival rates were significantly different between the ABC and GCB groups classified by the LIMD1-MYBL1 Index. In Scott *et al.* dataset, the performance of LIMD1-MYBL1 Index was satisfactory compared with the common IHC-based algorithms, and similar to the Nanostring-based Lymph2Cx assay.

In the next step, more research is needed in order to achieve a successful translation of the LIMD1-MYBL1 Index from Affymetrix microarray to real-time RT-PCR assays, thus allowing broader access and utilization in the clinical setting. In routine practice most diagnostic materials is formalin-fixed and paraffin embedded (FFPE), thus it is highly interesting to assess the utility of the LIMD1-MYBL1 Index in FFPE samples. Future translational research should focus on the development and validation of the real-time RT-PCR based LIMD1-MYBL1 Index using FFPE samples. In parallel, immunohistochemical analysis of the two gene products would also be interesting. Ultimately, a comparison of an immunophenotypic algorithm and a gene expression signature may help to determine the best platform for future clinical application.

The identification of two genes that associated with the disease subtypes and clinical outcomes may reveal targets for the development of therapy for DLBCL. To the best of our knowledge, *LIMD1* had not been reported as a specific marker for ABC-DLBCL. *LIMD1* is encoded at chromosome 3p21.3, a region that is commonly deleted in many solid malignancies^16^. *LIMD1* specifically interacts with pRB to repress E2F-mediated transcription and thus reduces cell proliferation in vitro and the incidence of lung metastases in vivo^17^. The loss of *LIMD1* expression, leading to the dysregulation of pRB and the cell cycle, may therefore be an early critical step in lung tumor development. It is intriguing that *LIMD1* was significantly overexpressed in ABC relative to the level in GCB. Based on previous findings for osteoblasts^18^,^19^, LIMD1 can act as a positive regulator of NF-kB by linking p62 and TRAF6 to form a p62/TRAF6/a-PKC complex. The overexpression of *LIMD1* may thus contribute to the constitutive activation of the NF-kB pathway in ABC-DLBCL.

*MYBL1* belongs to the Myb oncogene family of transcription factors which are involved in the regulation of the proliferation and differentiation of different hemopoietic cells^20^. *MYBL1* was specifically induced in proliferating centroblasts and rapidly down-regulated during centroblast differentiation to more mature B cells, suggesting that it might be a specific marker for proliferating centroblasts^21^. Interestingly, *MYBL1* is located in the chromosome region 8q22, which is involved in recurrent translocations in malignant lymphoma; therefore, *MYBL1* could be a candidate for involvement in such translocations^22^. The ectopic mRNA and protein expression of *MYBL1* has been observed in Burkett’s lymphoma, sIg^+^ B-acute lymphoblastic leukemia and some chronic lymphocytic leukemias^23^. *MYBL1* has been shown to effectively activate the *Bcl-2* promoter through a Cdx-binding site and thereby up-regulate Bcl-2 expression in DHL-4 cells^24^. The regulation of Mybl1 to c-myc was confirmed in murine B-cell lymphoma and was hypothesized to override the proapoptotic program of GC B-cells, thereby promoting malignant transformation^25^. On the basis of current findings, future research is needed to understand the molecular mechanisms of activated *LIMD1* and *MYBL1* expression in subtypes of DLBCL.

In conclusion, we identified and validated the LIMD1-MYBL1 Index as a composite marker for both the subtype classification and prognosis in DLBCL. Use of this two-gene index would allow right selection of subgroup of patients most likely to benefit from targeted therapy while avoiding other patients’ overtreatment. Although little is known about the oncogenic roles of *LIMD1* and *MYBL1*, our findings have the potential to open new avenues of research into the molecular mechanisms of DLBCL.

## Materials and Methods

### Sample selection and clinical information

We studied frozen specimens of DLBCL tissue from 88 Chinese patients who were diagnosed at Fudan University Shanghai Cancer Center (FDUSCC) between April 2005 and December 2009. There were 35 women and 53 men with a median age of 60.5 years (range, 15–86 years). Thirty-nine patients were younger than 60 years, and 49 patients were older than 60 years. Follow-up data were available for 78 patients who had received a variety of primary treatments. The median follow-up was 41.5 months (range, 1–68 months), with a 3-year overall survival of 66.7% for the entire group. This study was approved by the ethics committee of the Fudan University Shanghai Cancer Center, and was performed according to the Declaration of Helsinki. The written informed consent was obtained from all participants. The clinical information and gene expression data for Lenz *et al.* and Scott *et al.* studies were retrieved from the Gene Expression Omnibus database (Accession number: GSE10846 and GSE53786)^26^.

### Gene expression profiling and statistical analysis

Genome-wide gene expression profiling was performed with fresh-frozen samples using the U133plus2 microarrays (Affymetrix, CA), as previously described^8^. The microarray data were analyzed using R software and packages from the Bioconductor project^27^,^28^. Each dataset were individually normalized using the Robust Multi-chip Average (RMA) algorithm^29^. After normalization, probeset-level data were log2 transformed.

The gold standard method for COO classification were described by Wright *et al.* previously^5^. The 14 Affymetrix probesets were downloaded from the website of Lymphoma/Leukemia Molecular Profiling Project and were used to build a COO subtype classifier. The expression values of *LIMD1* and *MYBL1* was integrated with a similar Bayesian approach as described by Wright *et al.*^5^. The Bayesian classifier was trained using the DLBCL-1 data with known ABC or GCB labels, and directly applied to the test samples. A sample is classified as ABC or GCB subtype if the probability that it belongs to the ABC or GCB subgroup is greater than 80%; otherwise it is considered as unclassified type.

The prognostic ability of the gene signatures were quantified by calculating the “C-statistic” which estimated the probability that for a pair of randomly chosen comparable samples, the sample with the higher risk prediction will experience an event before the other sample or belongs to a higher binary class^30^. The actuarial probability of survival was determined using the Kaplan-Meier method, and differences were compared using the log rank test. A Cox proportional-hazards model was used for multivariate analysis. All significance tests were two-sided, and a *P* value less than 0.05 was considered significant.

## Author Contributions

Q.X, X.M and X.D wrote the main manuscript. Q.X, C.T, S.N, Q.W and W.S collected the data. X.Y, F.W and F.L performed the experiments. Q.X, C.T and S.N performed the statistical analysis and prepared figures. All authors reviewed the manuscript.

## Additional Information

**How to cite this article**: Xu, Q. *et al.* Identification and validation of a two-gene expression index for subtype classification and prognosis in Diffuse Large B-Cell Lymphoma. *Sci. Rep.* 5, 10006; doi: 10.1038/srep10006 (2015).

## Supplementary Material

Supplementary Table

## Figures and Tables

**Table 1:** Summary of DLBCL dataset.

**Study cohort**	**DLBCL-1**	**DLBCL-2**	**DLBCL-3**
No. of patients	414	88	68
Specimen type	Frozen	Frozen	Frozen, FFPE
Therapy	Mixture	Mixture	R-CHOP
End point	COO, OS	COO, OS	COO, OS
Median age (range)	63 (14-92)	61 (15-86)	61 (16-86)
COO, n(%)			
ABC-like	167(40)	32(36)	28(41)
GCB-like	183(44)	37(42)	30(44)
Unclassified	64(16)	19(22)	10(15)
Platform	HG-U133 Plus 2	HG-U133 Plus 2	HG-U133 Plus 2
*LIMD1* probe set	222762_x_at	222762_x_at	222762_x_at
*MYBL1* probe set	213906_at	213906_at	213906_at
Reference	Lenz *et al.*, 2008^8^		Scott *et al.*, 2014^12^

Abbreviation: FFPE, formalin-fixed, paraffin-embedded ; COO, cell-of-origin classification; OS, overall survival

**Table 2:** Multivariate logistic regression of prognostic parameters in DLBCL-1.

**Covariates**	**Categories**	**Hazard Ratio (95% CI)**	***P***
**LIMD1-MYBL1 Index with IPI score and treatment variable**			
IPI score	1 = low, 2 = low-intermediate, 3 = high-intermediate, 4 = high	2.1 (1.6–2.6)	<0.001
LIMD1-MYBL1 Index	GCB vs. ABC	2.1 (1.4–3.2)	<0.001
Treatment	R-CHOP vs. CHOP	2.3 (1.4–3.5)	<0.001

**LIMD1-MYBL1 Index with individual IPI constituent factors and treatment variable**			
Age	<60 vs. ≥60	2.4 (1.6–3.7)	<0.001
Ann Arbor stage	I-II vs. III-IV	1.3 (0.9–2.0)	0.23
ECOG performance status	<2 vs. ≥2	2.2 (1.2–4.1)	0.01
Extranodal sites	≤1 vs. >1	1.4 (0.6–3.3)	0.40
LDH	normal vs. high	2.3 (1.5–3.5)	<0.001
LIMD1-MYBL1 Index	GCB vs. ABC	2.2 (1.4–3.2)	<0.001
Treatment	R-CHOP vs. CHOP	2.0 (1.3–3.1)	0.002
