# Supplementary material for: Identification and validation of a two-gene expression index for subtype classification and prognosis in Diffuse Large B-Cell Lymphoma
Source: Sci Rep. 2015 May 5;5:10006. doi: 10.1038/srep10006 (PMC4419520; doi:10.1038/srep10006)
Supplement: Supplementary Table [file srep10006-s1.doc]

**Title**

Identification and validation of a two-gene expression index for subtype classification and prognosis in Diffuse Large B-Cell Lymphoma

**Short Title**

*LIMD1-MYBL1* Gene Expression Index in DLBCL

**Authors and Affiliations**

Qinghua Xu ^1-5^, Cong Tan ^1-3^, Shujuan Ni ^1-3^, Qifeng Wang ^1-3^, Fei Wu ^4,5^, Fang Liu ^4^, Xun Ye ^4,5^, Xia Meng ^4,5^, Weiqi Sheng ^1-3^, Xiang Du ^1-3^

^1^ Department of Oncology, Shanghai Medical College, Fudan University, Shanghai, China;

^2^ Department of Pathology, Fudan University Shanghai Cancer Center, Shanghai, China;

^3^ Institute of Pathology, Fudan University, Shanghai, China;

^4^ Fudan University Shanghai Cancer Center – Institut Mérieux Laboratory, Shanghai, China;

^5^ bioMérieux (Shanghai) Company Limited, Shanghai, China;

Corresponding author: Prof. Xiang Du, Department of Pathology, Fudan University Shanghai Cancer Center, 270 Dong An Road, Shanghai, 200032, China, phone number: 0086 21 64175590-8911, email: [dx2008cn@163.com](mailto:dx2008cn@163.com).

## Table S1: Associations between patients’ characteristics and subtype classification in DLBCL-2

| Variable | Pts | LIMD1-MYBL1 Index | | | *P-*value |
| --- | --- | --- | --- | --- | --- |
|  |  | ABC  (n = 32) | GCB  (n = 34) | Unclassified  (n = 22) |  |
| Age (yrs) |  |  |  |  |  |
| < 60 | 39 | 12(30.8%) | 19(48.7%) | 8 (20.5%) |  |
| ≥ 60 | 49 | 20(40.8%) | 15(30.6%) | 14(28.6%) | 0.22 |
| Gender |  |  |  |  |  |
| male | 53 | 19(35.8%) | 20(37.7%) | 14(26.4%) |  |
| female | 35 | 13(37.1%) | 14(40.0%) | 8 (22.9%) | 0.93 |
| Stage |  |  |  |  |  |
| I-II | 30 | 11(36.7%) | 10(33.3%) | 9 (30.0%) |  |
| III-IV | 19 | 10(52.6%) | 6 (31.6%) | 3 (15.8%) | 0.44 |
| NA | 39 |  |  |  |  |
| LDH level (U/L) | |  |  |  |  |
| < 300 | 42 | 15(35.7%) | 12(28.6%) | 15(35.7%) |  |
| ≥300 | 20 | 7 (35.0%) | 11(55.0%) | 2 (10.0%) | 0.05 |
| NA | 26 |  |  |  |  |
| Treatment |  |  |  |  |  |
| CHOP | 37 | 8 (21.6%) | 16(43.2%) | 13(35.1%) |  |
| R-CHOP | 32 | 12(37.5%) | 13(40.6%) | 7 (21.9%) | 0.28 |
| NA | 19 |  |  |  |  |
